# Forsythiaside A Alleviates Kidney Injury and Intestinal Epithelium Dysfunction in IgA Nephropathy by Inhibiting TLR4/NF‐κB Signaling

**DOI:** 10.1002/kjm2.70144

**Published:** 2025-11-28

**Authors:** Meng‐Si Li, Kai Liu

**Affiliations:** ^1^ Department of Nephrology China Resources & WISCO General Hospital, Affiliated to Wuhan University of Science and Technology Wuhan Hubei China

**Keywords:** forsythiaside A, IgA nephropathy, inflammation, intestinal barrier injury, TLR4/NF‐κB pathway

## Abstract

IgA nephropathy (IgAN), the most common form of glomerulonephritis, is a major and growing public health issue. It results from intestinal barrier dysfunction that leads to mesangial deposition of pathogenic galactose‐deficient IgA1 (Gd‐IgA1) and renal inflammation. This study aimed to investigate the therapeutic effects and associated mechanisms of forsythiaside A on intestinal barrier injury in IgAN in animal models. Rats were treated with bovine serum albumin (BSA), carbon tetrachloride (CCl_4_), and lipopolysaccharide (LPS) to induce IgAN, followed by intragastric administration of forsythiaside A once daily from weeks 15 to 20 after model establishment. Biochemical markers, including 24‐h urinary protein, blood urea nitrogen (BUN), serum creatinine (SCr), renal and intestinal tissue pathology, and levels of pro‐inflammatory cytokines in the serum, kidney, and intestine, intestinal tight junction proteins, and TLR4/NF‐κB pathway components were examined. The results showed that forsythiaside A decreased 24‐h urinary protein, BUN, and SCr levels, alleviated renal damage, and attenuated glomerular and tubular lesions, collagen deposition, and glomerular IgA deposition in IgAN rats. Forsythiaside A treatment inhibited CD68‐positive macrophage infiltration in renal tissues and downregulated serum and renal levels of IL‐1β, IL‐6, and TNF‐α, while also alleviating intestinal barrier injury and intestinal inflammation, as shown by reduced levels of IL‐1β, IL‐6, and TNF‐α and increased expression of the intestinal tight junction proteins occludin and ZO‐1. Lastly, forsythiaside A treatment lowered serum LPS concentrations, as well as renal and intestinal levels of TLR4, p‐NF‐κB p65, and p‐IκBα, and raised both renal and intestinal levels of IκBα. Collectively, forsythiaside A was found to ameliorate the progression of IgAN in rats by alleviating inflammation and intestinal barrier injury by suppression of TLR4/NF‐κB signaling.

## 1 Introduction

Immunoglobulin A (IgA) nephropathy (IgAN) is a primary glomerular disease characterized by the dominant or codominant mesangial deposition of IgA [1]. The diagnosis of IgAN can only be confirmed by renal biopsy, with pathological changes including mesangial cell proliferation, tubular atrophy, interstitial fibrosis, glomerular segmental sclerosis, and capillary proliferative lesions [2]. The clinical manifestations involve varying degrees of proteinuria together with recurrent gross or microscopic haematuria, with some patients developing severe hypertension or renal insufficiency [3]. Evidence suggests variations in the geographical distribution of IgAN, with the greatest prevalence seen in Asia, followed by Europe and Africa [4]. It is estimated that in up to 40% of patients, IgAN will progress to end‐stage renal disease (ESRD) within 20 years of diagnosis and usually requires kidney replacement, involving both heavy financial burdens and physical pain and suffering [5]. Inhibitors of the renin‐angiotensin system (RAS) are the first choice for non‐immunological treatment of IgAN. However, their prolonged use can lead to aldosterone escape, which greatly offsets the therapeutic benefits of RAS blockers and is associated with various health problems [6]. Besides, despite the efficacy of glucocorticoids or immunosuppressants in controlling proteinuria in patients with IgAN, their use is frequently accompanied by side effects, such as the occurrence of peptic ulcers, osteoporosis, and rash [7, 8]. Hence, the development of safer and more effective treatment approaches for patients with IgAN has become particularly important.

Although the exact pathogenesis of IgAN has not been elucidated to date, it is well‐recognized that patients with the disease have high levels of galactose‐deficient IgA1 (Gd‐IgA1) in their circulation and glomerular deposits [9]. Gd‐IgA1 is an autoantigen and triggers the production of autoantibodies, which combine with Gd‐IgA1 to form immune complexes. These complexes are deposited in glomerular mesangial areas, leading to activation of the complement system and release of inflammatory factors, damaging the renal tissue and eventually resulting in the onset and progression of IgAN [10]. IgA is produced primarily by mucosa‐associated lymphoid tissues (MALTs), mostly within the gastrointestinal tract. Several lines of evidence suggest that IgA produced by the intestinal mucosa is the primary source of pathogenic Gd‐IgA1 in IgAN [11]. It has been reported that damage to the intestinal barrier induces abnormal microbial responses, increases antigen absorption, and activates MALTs to produce Gd‐IgA1 [12]. In addition, impairment of the intestinal barrier leads to increased permeability of the intestinal mucosa, leading to the translocation of bacteria and endotoxins from the intestinal tract to the blood and lymphatic circulation, and in turn inducing the overproduction of inflammatory mediators and consequent damage to renal tissues [13]. A genome‐wide association study of IgAN in 20,612 patients identified a number of loci linked to IgAN development, including genes associated with maintenance of the integrity of the intestinal epithelial barrier, IgA synthesis in the gut, mucosal responses to pathogens, and the development of inflammatory bowel disease [14]. Accordingly, the alleviation of intestinal barrier dysfunction might contribute to impeding the development and aggravation of IgAN.

Due to their unique advantages in disease prevention, treatment, and rehabilitation, traditional Chinese medicines are valuable sources of novel drugs with significant therapeutic efficacy, with important contributions to human health. The herb Forsythiae Fructus has been widely used in the clinical treatment of infectious diseases, fever, and inflammation for thousands of years [15]. Forsythiaside A is a major bioactive component of Forsythiae Fructus and recent pharmacological studies have confirmed its anti‐inflammatory, antibacterial, hepatoprotective, antioxidant, neuroprotective, and antiviral properties [16]. Furthermore, the protective effects of forsythiaside A against renal and intestinal injury have been demonstrated in many studies. For example, forsythiaside A has been shown to reduce proteinuria, serum creatinine (SCr), and blood urea nitrogen (BUN) levels, as well as mitigating oxidative stress and the inflammatory response, thereby alleviating adriamycin‐induced nephropathy in rats [17]. Forsythiaside A has also been found to prevent sepsis‐induced acute kidney injury in mice by inhibiting renal inflammation and apoptosis [18], and can also ameliorate inflammation and methotrexate‐induced damage to the small intestine in rats through suppression of the NLRP3 pathway [19]. Lung and colon inflammation and damage to the epithelial barrier caused by intratracheal injection of lipopolysaccharide (LPS) in mice can also be alleviated by forsythiaside A treatment [20].

However, to our knowledge, the effects of forsythiaside A on IgAN progression remain unknown. This study was designed to investigate whether forsythiaside A can ameliorate intestinal barrier dysfunction and prevent renal injury in rat models of IgAN. The mechanism underlying the action of forsythiaside A was explored by evaluation of the expression of the TLR4/NF‐κB signaling pathway in the rats.

## 2 Materials and Methods

### 2.1 IgAN Rat Model and Experimental Protocols

Four‐week‐old Sprague–Dawley rats (180–220 g) were acquired from SPF Animals Biotechnology (Beijing, China) and were housed under standard laboratory conditions (25°C, 50% relative humidity, and a 12‐h light/dark cycle). After acclimatization for 1 week, the experimental IgAN model was induced, followed by drug administration as described below. All animal experiments received ethical approval from the Institutional Animal Care and Use Committee of Wuhan Myhalic Biotechnology Co. Ltd. (approval number: HLK‐202403057; approval date: March 5, 2024).

The rats were randomly assigned to six groups, namely, the normal, IgAN model, IgAN + low dosage forsythiaside A (20 mg/kg), IgAN + medium dosage forsythiaside A (40 mg/kg), IgAN + high dosage forsythiaside A (80 mg/kg), and IgAN + Losartan groups, with 8 rats in each group. To induce IgAN, the rats were given oral doses of 600 mg/kg bovine serum albumin (BSA; #ST2249; Beyotime, Shanghai, China) on alternate days, as well as subcutaneous injections of 0.5 mL castor oil (#HY‐107799; MedChemExpress, Shanghai, China) containing 0.1 mL carbon tetrachloride (CCl_4_; #HY‐Y0298; MedChemExpress) once a week and intravenous injections (via the tail vein) of 0.25 mg/kg LPS (#ST1470; Beyotime) on weeks 7, 10, and 13, for an overall period of 14 weeks [21]. Rats in the drug treatment groups received intragastric administration of forsythiaside A (#HY‐N0028; MedChemExpress) once a day starting from week 15 until week 20. Losartan (30 mg/kg/day; #HY‐17512; MedChemExpress) was given to rats as the pharmacological control. The dosages of forsythiaside A [20] and losartan [21] were selected according to previous studies. Rats in the normal and model groups received daily doses of the same volume of vehicle (normal saline) by intragastric administration. The 24‐h urine samples were collected weekly starting from week 15 to measure urinary protein levels. Blood samples were collected from the aorta at the end of the experiment for the measurement of biochemical parameters. The right kidneys of the rats as well as a portion of the intestinal tissue were stored at −80°C for subsequent ELISA, RT‐qPCR, and western blotting, while the left kidneys and remaining portions of intestinal tissues were fixed in 4% paraformaldehyde for histological, immunofluorescence, and immunohistochemical staining.

### 2.2 Biochemical Assays

Blood samples were centrifuged at 3000×*g* for 10 min at 4°C to obtain the serum, which was immediately stored at −80°C. The 24‐h urine protein, as well as serum levels of BUN and SCr, were measured using an automatic biochemical analyzer (Hitachi 7080, Japan).

### 2.3 ELISA

Rat intestinal tissues were homogenized in RIPA buffer (#R0010; Reanta, Beijing, China) and centrifuged at 12000×*g* for 20 min at 4°C to isolate the supernatant. The levels of IL‐1β (#ER1094), IL‐6 (#ER0042), TNF‐α (#ER1393), and LPS (#EU3126) in the sera and supernatants were assayed by commercially available ELISA kits (FineTest, Wuhan, China), following the kit instructions.

### 2.4 RT‐qPCR

Total RNA was extracted from rat renal and intestinal tissues using TRIzol reagent (#R1030; APPLYGEN, Beijing, China) and the quality and concentration of the RNA were verified using a UV spectrophotometer. A Quantscript RT kit (#KR103; Tiangen, Beijing, China) was used for the reverse transcription of 1 μg of total RNA. qPCR was performed using a SuperReal PreMix Plus (SYBR Green) kit (#FP215; Tiangen) and specific primers (Table 1) on a Light Cycler96 system (Roche, Mannheim, Germany). All mRNA levels were calculated using the 2^−ΔΔCt^ method, with normalization to GAPDH.

**TABLE 1:** Primer sequences used for RT‐qPCR.

Gene	Primer sequences
IL‐1β	Forward: 5′‐GAAGTCAAGACCAAAGTGG‐3′
	Reverse: 5′‐TGAAGTCAACTATGTCCCG‐3′
IL‐6	Forward: 5′‐AGTTGCCTTCTTGGGACTGA‐3′
	Reverse: 5′‐CCTCCGACTTGTGAAGTGGT‐3′
TNF‐α	Forward: 5′‐ACTCCCAGAAAAGCAAGCAA‐3′
	Reverse: 5′‐CGAGCAGGAATGAGAAGAGG‐3′
Occludin	Forward: 5′‐GACCTTGTCCGTGGATGACTTCAG‐3′
	Reverse: 5′‐ATCAGCAGCAGCCATGTACTCTTC‐3′
ZO‐1	Forward: 5′‐CACACGATGCTCAGAGACGAAGG‐3′
	Reverse: 5′‐CTGTATGGTGGCTGCTCAAGGTC‐3′
GAPDH	Forward: 5′‐GGGCAGCCCAGAACATCAT‐3′
	Reverse: 5′‐CCAGTGAGCTTCCCGTTCAG‐3′

### 2.5 Western Blotting

RIPA buffer containing a mixture of protease and phosphatase inhibitors (#P1261; APPLYGEN) was used to lyse renal and intestinal tissues. The lysates were then centrifuged at 12000×*g* for 20 min at 4°C and the protein concentration in the resulting supernatant was measured using a Bradford protein assay kit (#P1510; APPLYGEN). Then, 20 μg of protein from the samples were mixed with SDS‐PAGE loading buffer (#B1007; APPLYGEN), heated for 5 min at 95°C, separated on SDS‐PAGE, and transferred to polyvinylidene fluoride (PVDF) membranes (0.45 μm). After blocking for 1 h at room temperature with 5% nonfat milk, the membranes were incubated with primary (overnight, 4°C) and secondary (2 h, 25°C) antibodies. The primary antibodies anti‐IL‐1β (#AF5103; 1/1000), anti‐IL‐6 (#DF6087; 1/1000), anti‐TNF‐α (#AF7014; 1/500), anti‐occludin (#AF4605; 1/1000), anti‐ZO‐1 (#AF5145; 1/1000), anti‐TLR4 (#AF7017; 1/1000), anti‐NF‐κB p65 (#AF5006; 1/1000), anti‐p‐NF‐κB p65 (#AF2006; 1/1000), anti‐p‐IκBα (#AF2002; 1/1000), anti‐IκBα (#AF5002; 1/1000), and anti‐GAPDH (#AF0911; 1/3000), and the secondary antibody anti‐IgG (#S0001; 1/3000) were all purchased from Affinity Bioscience (Jiangsu, China). The immunoreactive bands were detected using the Super ECL Plus reagent (#P1050; APPLYGEN) and quantified by densitometry using ImageJ 6.0 software. The values were normalized to those of the loading control GAPDH, IκBα for p‐IκBα, and NF‐κB p65 for p‐NF‐κB p65.

### 2.6 Pathological Assessment

Rat kidney and ileum tissues were fixed with 4% paraformaldehyde, dehydrated in an alcohol gradient, embedded in paraffin, and cut into 5‐μm sections. Following deparaffinization in xylene and rehydration in decreasing concentrations of alcohol, the sections were stained with either hematoxylin and eosin (HE; #C1410; APPLYGEN) or Masson's trichrome stain (#B1130; APPLYGEN) using kits as directed. The stained sections were observed and imaged under a light microscope (BX51; Olympus, Tokyo, Japan). Renal tissues were evaluated and scored as previously described [22]. The collagen volume fraction (%) in images of Masson‐stained sections of renal tissues and villus lengths in images of HE‐stained ileal tissues were determined using ImageJ 6.0 software.

### 2.7 Immunofluorescence and Immunohistochemistry Staining

Paraffin‐embedded sections (5‐μm thick) of rat kidney and ileal tissues were dewaxed and dehydrated in an ethanol gradient. Thereafter, the sections were heated in sodium citrate solution for antigen retrieval, followed by inactivation of endogenous peroxidases with 3% hydrogen peroxide and blocking of nonspecific binding with 5% normal goat serum. For immunofluorescence analysis, the sections were incubated with the primary antibodies anti‐IgA (#SM1027P; 1/100; Origene, USA), anti‐occludin (#DF7504; 1/200; Affinity Bioscience), anti‐ZO‐1 (#AF5145; 1/200; Affinity Bioscience), and anti‐CD68 (#DF7518; 1/100) overnight at 4°C, followed by FITC‐labeled (green) (#S0008; 1/100; Affinity Bioscience) and Cy3‐labeled (#S0011; 1/100; Affinity Bioscience) secondary antibodies for 1 h at room temperature. Finally, an antifluorescent quencher containing 4′,6‐diamidino‐2‐phenylindole (DAPI) (#A598329; Aladdin, Shanghai, China) was used to counterstain the nuclei (blue), the slides were mounted, and evaluated and imaged using a fluorescence microscope (BX63; Olympus). ImageJ 6.0 software was employed to quantify the average fluorescence intensity. For immunohistochemical analysis, an anti‐CD68 primary antibody (#DF7518; 1/100) and HRP‐labeled secondary antibody (#S0001; 1/200) were used for successive treatment of the sections; both antibodies were acquired from Affinity Bioscience. After addition of the DAB staining solution (#B1072; APPLYGEN) for color development, the sections were counterstained with hematoxylin. Images were captured under a light microscope (BX51; Olympus). Quantitative analysis of the positive area was performed using ImageJ 6.0 software.

### 2.8 Statistical Analysis

SPSS version 25.0 software was used for data analysis. All data are presented as the mean ± SD. One‐way ANOVA followed by the Bonferroni correction was used for comparison of multiple groups. The graphs were created using GraphPad Prism version 6.0 software. *p* < 0.05 was considered statistically significant.

## 3 Results

### 3.1 Forsythiaside A Ameliorates Renal Function and Pathological Changes in IgAN Rats

**FIGURE 1:**
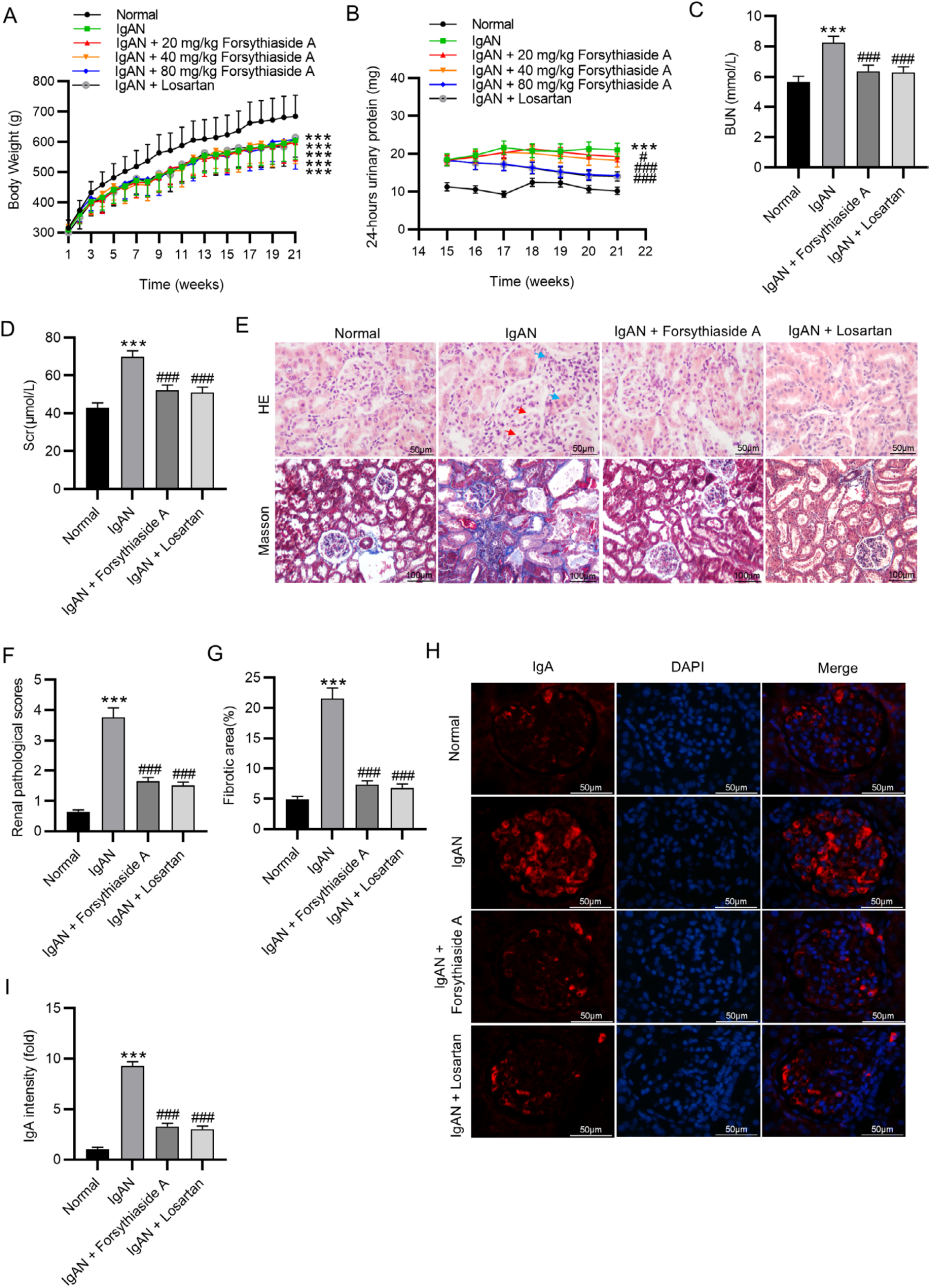
Forsythiaside A ameliorates renal dysfunction and pathological changes in IgAN model rats. (A) Changes in rat body weight over the entire experimental period. (B) 24‐h urinary protein levels in rats during the drug treatment period. (C, D) BUN and SCr levels in rats at the end of the experiment. (E) Representative HE‐ (scale bar: 50 μm) and Masson (scale bar: 100 μm)‐stained images showing renal histopathological changes. Red arrows: Mesangial cell proliferation; Blue arrows: Renal tubular atrophy and interstitial fibrosis. (F) Quantification of renal pathological scores based on the HE‐staining results in (E). (G) Quantification of the fibrotic area based on the Masson‐staining results in (E). (H) Representative immunofluorescence images showing glomerular deposition of IgA. Scale bar: 50 μm. (I) IgA fluorescence intensity based on the staining results in (H). *N* = 8. ***p < 0.001 versus normal group; ^#^ *p* < 0.05, ^###^ *p* < 0.001 versus IgAN group.

As shown in Figure 1A, the IgAN model rats showed obvious weight loss. The body weights were not restored after treatment with losartan or different doses of forsythiaside A. The IgAN rats developed significant proteinuria, a typical symptom of IgAN, shown by increased 24‐h urinary protein levels compared with normal rats. Treatment with varying doses of forsythiaside A effectively reduced the urinary protein levels in IgAN rats, with the greatest effects seen at higher dosages (Figure 1B). Notably, the 80 mg/kg dose of forsythiaside A showed the most potent effects and was closest to the effects of losartan. Accordingly, 80 mg/kg forsythiaside A was used to treat the IgAN rats in subsequent experiments. Compared with normal rats, the IgAN rats exhibited renal insufficiency, evident in the marked elevations of both BUN and SCr levels. However, both forsythiaside A and losartan were found to attenuate BUN and Scr levels and improve kidney function in the IgAN rats (Figure 1C,D). The observed histopathological changes corresponded to the changes in biochemical markers. Mild to moderate proliferation of glomerular mesangial cells, accumulation of the mesangial matrix, thickening of the glomerular basement membrane, and partial swelling of renal tubular epithelial cells were observed in the renal tissues of IgAN rats, as shown by HE staining. In contrast, treatment with either forsythiaside A or losartan largely alleviated these glomerular and tubular lesions. Masson staining indicated that the IgAN‐induced deposition of collagen in the rat kidneys was ameliorated by either forsythiaside A or losartan treatment (Figure 1E). Quantitative analyzes of HE‐ and Masson‐stained tissues showed that treatment with forsythiaside A or losartan reverses increases in both the renal pathological score and fibrotic area caused by IgAN (Figure 1F,G). Additionally, the immunofluorescence results revealed marked glomerular deposition of IgA in the IgAN model rats, while treatment with either forsythiaside A or losartan reduced the IgA fluorescence intensity (Figure 1H,I).

### 3.2 Forsythiaside A Attenuates Renal Inflammation in IgAN Rats

**FIGURE 2:**
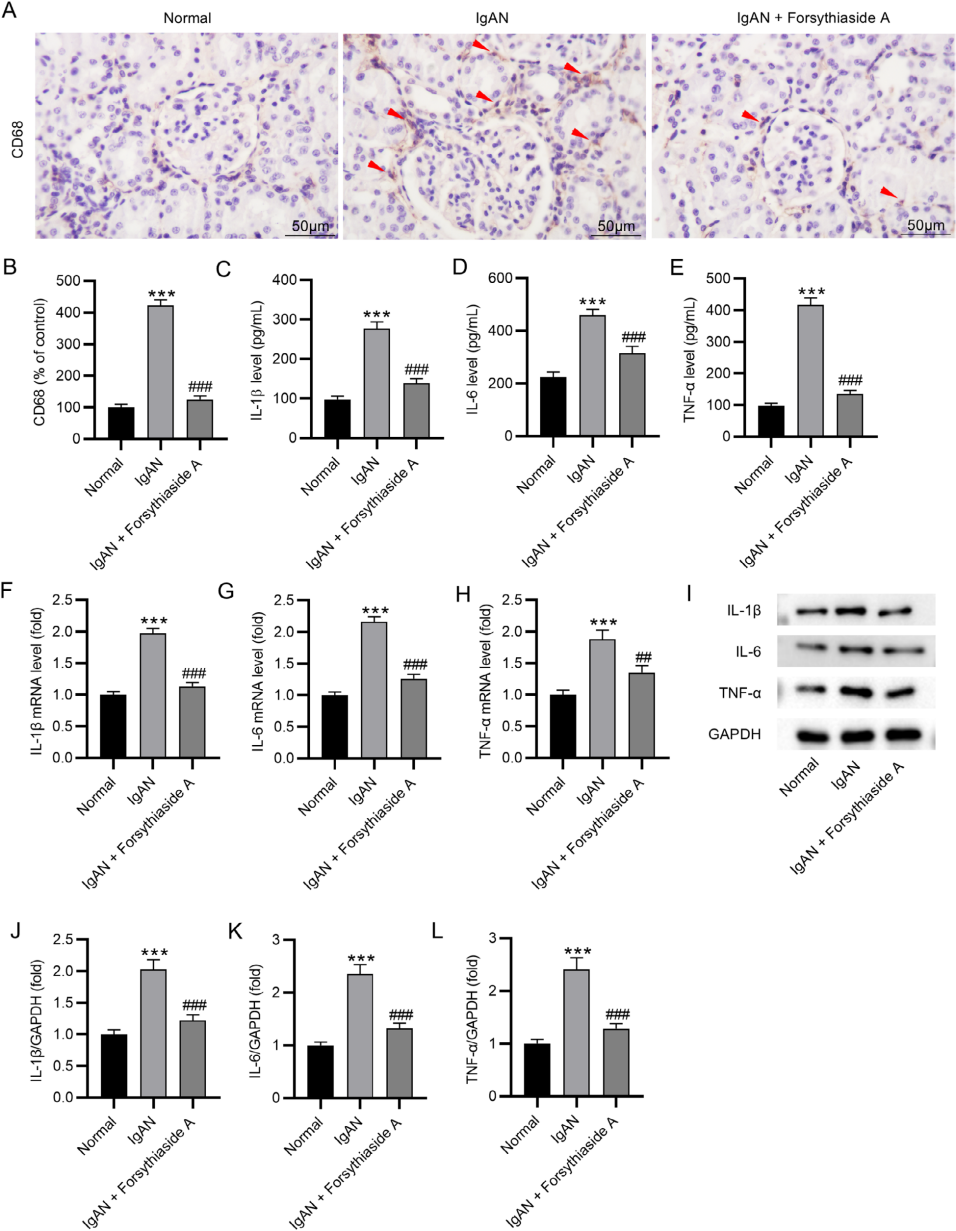
Forsythiaside A attenuates renal inflammation in the IgAN model rats. (A) Representative images of immunohistochemical staining of CD68‐positive macrophages in rat kidney tissues. Scale bar: 50 μm. (B) Percentage of CD68‐positive cells calculated from (A). (C–E) ELISA measurements of IL‐1β, IL‐6, and TNF‐α levels in rat sera. (F–H) RT‐qPCR measurements of IL‐1β, IL‐6, and TNF‐α mRNA levels in rat renal tissues. (I–L) Western blotting showing IL‐1β, IL‐6, and TNF‐α protein levels in rat renal tissues. *N* = 8. ***p < 0.001 versus normal group; ^##^ *p* < 0.01, ^###^ *p* < 0.001 versus IgAN group.

Immunohistochemical staining of rat renal tissues using an anti‐CD68 antibody showed marked increases in the number of CD68‐positive macrophages in the kidneys of IgAN rats, with scattering of the macrophages throughout both the glomeruli and tubulointerstitium. In contrast, infiltration of CD68‐positive macrophages was markedly suppressed after treatment with forsythiaside A relative to the model rats (Figure 2A,B). The anti‐inflammatory effects of forsythiaside A were further confirmed by measuring the levels of pro‐inflammatory cytokines. The ELISA results showed that the serum concentrations of IL‐1β, IL‐6, and TNF‐α in the IgAN rats were considerably higher than those in the normal rats, while forsythiaside A treatment effectively reduced these levels (Figure 2C–E). Consistently, IgAN‐induced increases in the mRNA and protein levels of IL‐1β, IL‐6, and TNF‐α in rat renal tissues were reversed after forsythiaside A treatment (Figure 2F–L).

### 3.3 Forsythiaside A Alleviates Intestinal Barrier Injury in IgAN Rats

**FIGURE 3:**
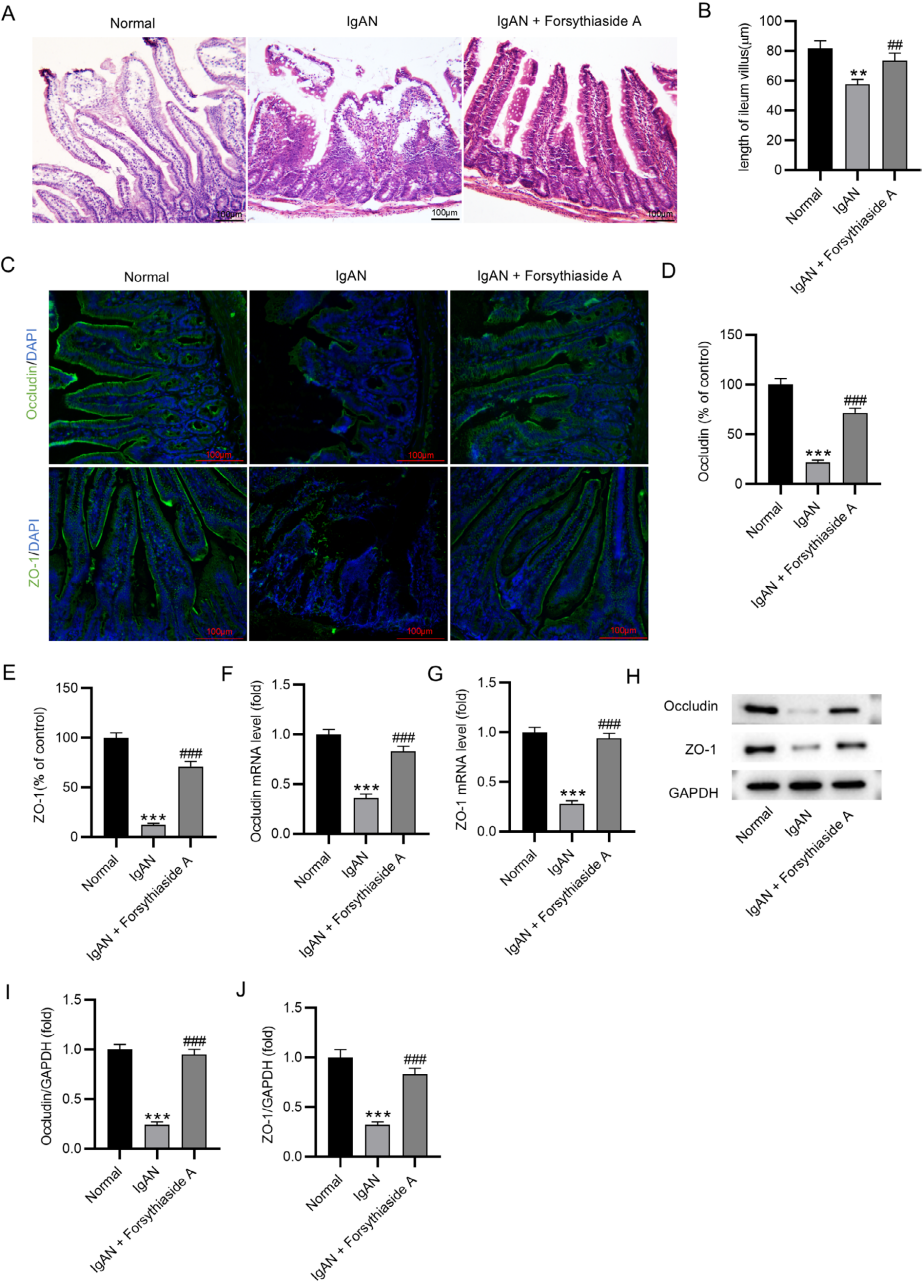
Forsythiaside A alleviates intestinal barrier injury in IgAN model rats. (A) Representative images of HE‐stained ileal tissues showing histopathological changes. Scale bar: 100 μm. (B) Quantification of villus lengths in ileal tissues from (A). (C) Representative images of immunohistochemical analysis of occludin and ZO‐1 expression in rat ileal tissues. Scale bar: 100 μm. (D, E) Quantification of occludin and ZO‐1 expression based on (A). (F, G) RT‐qPCR measurement of occludin and ZO‐1 mRNA levels in rat intestinal tissues. (H–J) Western blotting showing occludin and ZO‐1 protein levels in rat intestinal tissues. *N* = 8. **p < 0.01, ***p < 0.001 versus normal group; ^##^ *p* < 0.01, ^###^ *p* < 0.001 versus IgAN group.

Rat ileal tissues were stained with HE to assess histopathological changes. Tissues from the IgAN group showed disordering of the intestinal villi, with reduced villus heights and increased crypt depths relative to the normal group. These changes were reversed by forsythiaside A treatment (Figure 3A,B). The expression of intestinal tight junction proteins was assessed to determine whether forsythiaside A can protect against intestinal barrier dysfunction in IgAN rats. Immunofluorescence staining of ileal tissues showed significantly reduced occludin‐ and ZO‐1‐positive areas compared to the normal group. These changes, however, were reversed after treatment with forsythiaside A (Figure 3C–E). In addition, RT‐qPCR and western blotting further demonstrated that forsythiaside A increased the mRNA and protein levels of occludin and ZO‐1 relative to the model rats (Figure 3F–J).

### 3.4 Forsythiaside A Suppresses Intestinal Inflammation in IgAN Rats

**FIGURE 4:**
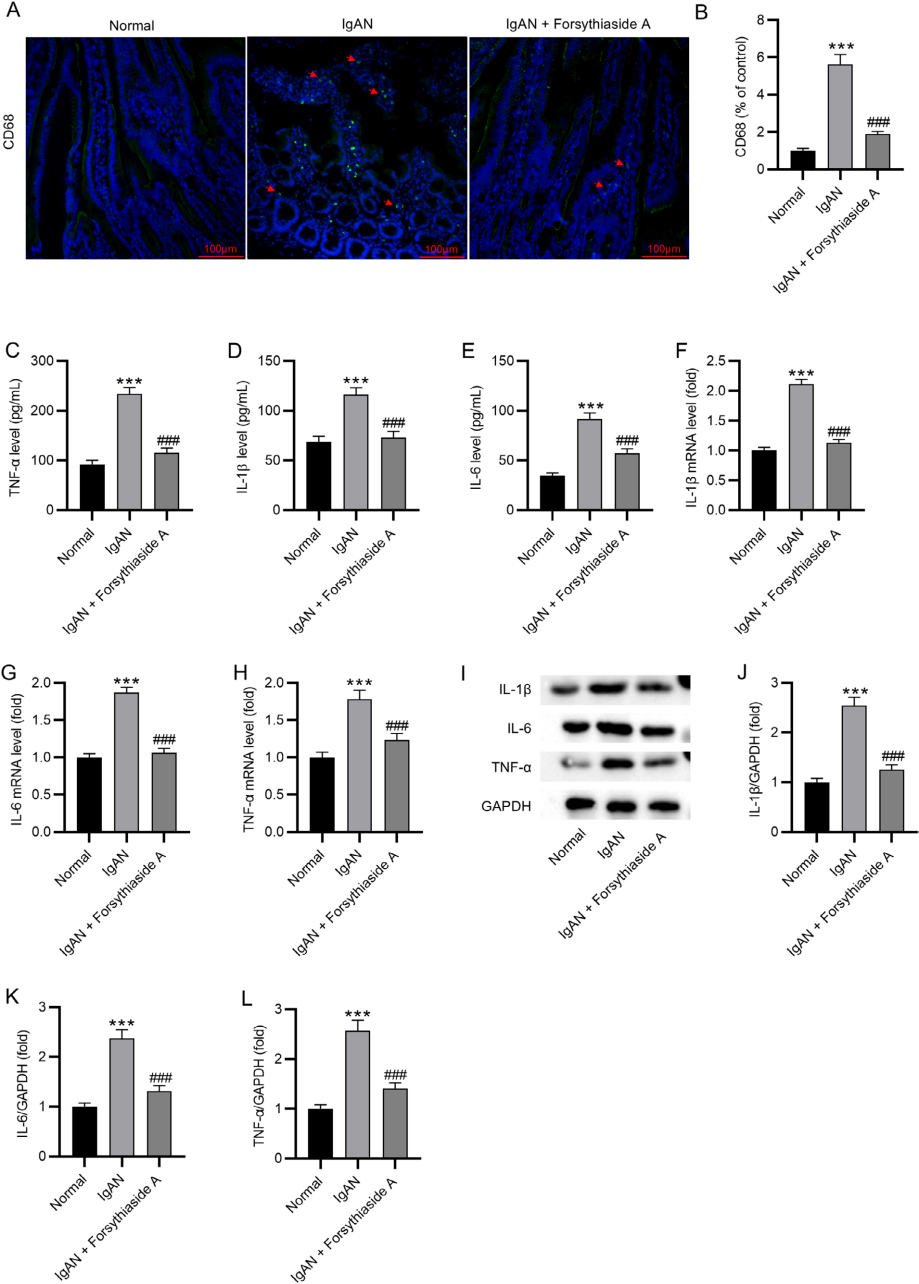
Forsythiaside A suppresses intestinal inflammation in IgAN model rats. (A) Representative images of immunofluorescence staining of CD68 in rat intestinal tissues. Scale bar: 100 μm. (B) Determination of CD68‐positive cells (%) based on staining results in (A). (C–E) ELISA measurements of IL‐1β, IL‐6, and TNF‐α levels in intestinal tissue homogenates of rats. (F–L) RT‐qPCR analysis of IL‐1β and IL‐6 mRNA levels in rat intestinal tissues. *N* = 8. ***p < 0.001 versus normal group; ^###^ *p* < 0.001 versus IgAN group.

Similarly, immunofluorescence staining for CD68 in rat intestinal tissues showed that the IgAN‐induced increase in the number of CD68‐positive macrophages was suppressed by forsythiaside A treatment (Figure 4A,B). The levels of key inflammatory cytokines in rat intestinal tissues were then measured to confirm the effects of forsythiaside A on intestinal inflammation in IgAN rats. The ELISA results showed that IL‐1β, IL‐6, and TNF‐α levels in intestinal tissue homogenates from IgAN rats were significantly raised compared with those from normal rats, while these effects were reversed following treatment with forsythiaside A (Figure 4C–E). These results were confirmed by the measurement of IL‐1β, IL‐6, and TNF‐α mRNA and protein expression in the intestinal tissues (Figure 4F–L).

### 3.5 Forsythiaside A Inhibits TLR4/NF‐κB Pathway Activation in IgAN Rats

**FIGURE 5:**
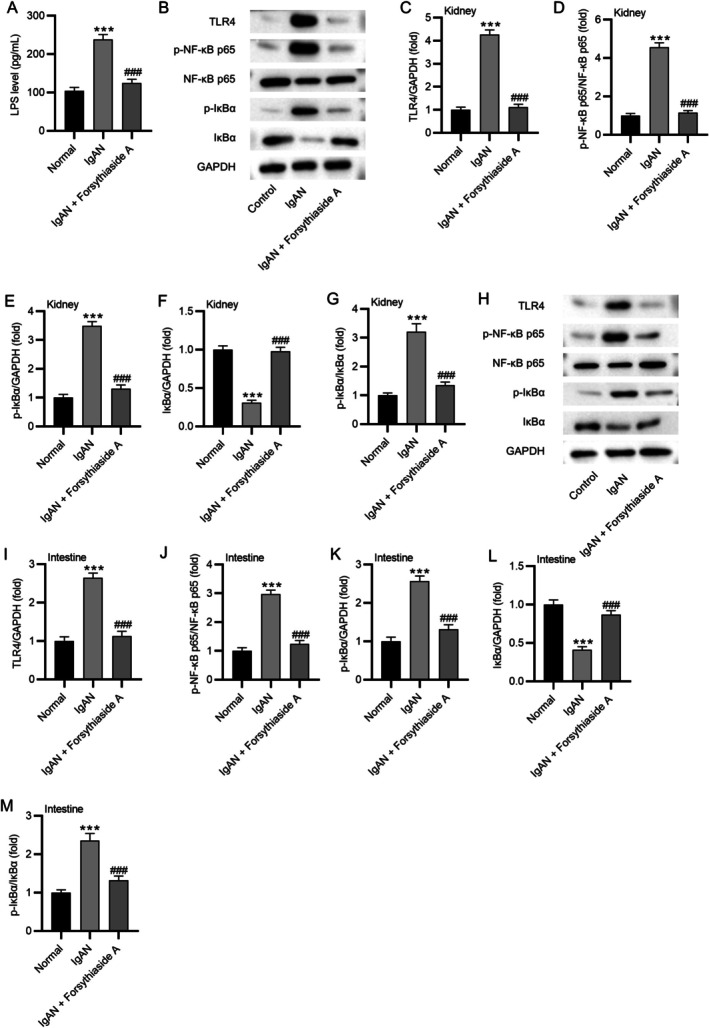
Forsythiaside A inhibits TLR4/NF‐κB pathway activation in IgAN model rats. (A) ELISA measurement of LPS contents in rat sera. (B–G) Western blotting showing TLR4, p‐NF‐κB p65, NF‐κB p65, p‐IκBα, and IκBα protein levels in rat renal tissues. (H–M) Western blotting showing TLR4, p‐NF‐κB p65, NF‐κB p65, p‐IκBα, and IκBα protein levels in rat intestinal tissues. *N* = 8. ***p < 0.001 versus normal group; ^###^ *p* < 0.001 versus IgAN group.

Intestinal barrier injury caused by IgAN led to increased production of LPS (also known as endotoxin), as evidenced by the marked increase in serum LPS content in the IgAN model group versus the normal group. However, LPS secretion was markedly inhibited after forsythiaside A treatment (Figure 5A). LPS is recognized by TLR4, leading to activation of the NF‐κB pathway and subsequent inflammation. Western blotting indicated that the protein levels of TLR4, p‐NF‐κB p65, and p‐IκBα were upregulated while those of IκBα were downregulated in both renal and intestinal tissues of IgAN rats. The changes in the expression of the TLR4/NF‐κB pathway‐related molecules were reversed by forsythiaside A treatment (Figure 5B–M).

## 4 Discussion

IgAN is a common form of primary glomerulonephritis throughout the world and represents a major cause of ESRD. Patients with ESRD require kidney transplantation or long‐term dependence on dialysis to sustain life. RAS blockers, steroids, and immunosuppressants are often used for IgAN treatment, despite reductions in their effectiveness due to significant side effects. In recent decades, the beneficial effects of herbal medicines and their active constituents in balancing immune function, alleviating inflammation, reducing proteinuria, protecting renal function, and slowing the progression of IgAN have been confirmed. In the present study, the therapeutic effects of forsythiaside A, the major active component of Forsythiae Fructus, and its associated mechanism in IgAN treatment were assessed in rat models.

The levels of proteinuria and parameters of renal function were first analyzed in the IgAN rat models with and without administration of forsythiaside A. Proteinuria is a major clinical manifestation of IgAN, and is both independently predictive of the glomerular filtration rate and a key factor in determining the prognosis of IgAN. Previous studies have reported the efficacy of forsythiaside A in ameliorating both proteinuria and renal dysfunction [17, 18]. The present results showed that forsythiaside A significantly reduced increases in proteinuria, BUN, and Scr levels in IgAN rats. Clinically, impairment of kidney function is closely related to structural damage to glomerular, tubular, and interstitial tissues in IgAN [23]. Histological analysis showed that forsythiaside A treatment reduced the proliferation of glomerular mesangial cells and expansion of the extracellular matrix, as well as the degree of glomerular basement membrane thickening, tubulointerstitial fibrosis, and IgA deposition in IgAN rats, consistent with the improvements in renal function induced by forsythiaside A.

Evidence suggests that inflammation represents the essential driving force behind the progression of IgAN. Abnormal immune responses can result in the deposition of large amounts of circulating Gd‐IgA1‐IgG/IgA complexes in the glomerular mesangial area, leading to increased production of inflammatory mediators such as IL‐1β, IL‐6, and TNF‐α and triggering an inflammatory response. Persistent inflammation alters glomerular permeability, followed by the development of proteinuria and tubulointerstitial injury. Clinical observation has shown markedly higher levels of inflammatory factors in the urine of patients with IgAN, which have been linked to poor prognosis in these patients [24]. Furthermore, significant increases in inflammatory cytokine levels have been found in the sera and renal tissues of IgAN animal models [25, 26]. Shuiai et al. reported that renal tubular epithelial cells from patients with IgAN secrete large amounts of MCP‐1, a major chemokine responsible for monocyte/macrophage chemotaxis and activation [27]. MCP‐1 induces the infiltration of monocytes and macrophages in the renal tubules and interstitium, leading to the production of various pro‐fibrotic and pro‐inflammatory cytokines, and exacerbating tubulointerstitial lesions and kidney dysfunction in patients with IgAN. It is well‐documented that the anti‐inflammatory effects of forsythiaside A in experimental models of human diseases involve inhibiting the production and expression of inflammatory cytokines and chemokines [28, 29]. The present results indicated that forsythiaside A treatment inhibited the infiltration of CD68‐positive macrophages in both the glomeruli and tubulointerstitium, and also reduced the levels of IL‐1β, IL‐6, and TNF‐α in the sera, kidneys, and intestines of IgAN rats.

The intestinal epithelial barrier is composed of epithelial cells, intercellular tight junctions, and a mucous layer. The structural and functional integrity of the barrier contributes to the body's defense against harmful pathogens and the maintenance of normal immune functions. Recent studies have reported associations between impairment of the intestinal barrier and the development of multiple intestinal or extra‐intestinal disorders, including immune‐related kidney diseases such as IgAN, lupus nephritis, and diabetic nephropathy [30, 31]. Disruptions of intestinal barrier integrity can cause leukocyte infiltration, stimulate the release of pro‐inflammatory cytokines and subsequent local inflammation, and promote endocytosis of tight junction proteins, all of which exacerbate the permeability of the intestinal mucosa. Increased permeability of the intestinal barrier can lead to the translocation of harmful bacteria from the gut to the systemic circulation, ultimately aggravating renal inflammation. Additionally, intestinal barrier dysfunction has been found to induce the development of IgAN through activation of MALTs and stimulating Gd‐IgA1 production. In a prospective cross‐sectional pilot study conducted by Seikrit et al., permeability in the small intestine was observed to be increased in most patients with IgAN and non‐IgAN glomerulopathies, suggesting an association between intestinal barrier dysfunction and IgAN development [32]. Tight junctions are important junctional complexes between epithelial cells, and consist of both transmembrane proteins (occludin and claudin) and submembranous proteins (ZO proteins), functioning as a paracellular permeability barrier and maintaining cell polarities. It has been shown that reduced expression and translocation of tight junction proteins can disrupt the integrity of the barrier, increase the permeability of the intestinal epithelium, and lead to both inflammation and damage to the intestinal mucosa [33]. Decreased expression of tight junction proteins has been observed in animal models of IgAN [34, 35]. Previously, forsythiaside A was reported to ameliorate CCl_4_‐induced intestinal damage and restore intestinal barrier function, shown by increased expression of ZO‐1, claudin‐1, and occludin in mouse ileal tissues [36]. The present study found that forsythiaside A treatment increased both occludin and ZO‐1 levels in the intestinal tissues of IgAN rats, suggesting that forsythiaside A can maintain the integrity of the intestinal barrier.

The term endotoxin typically refers to LPS, a component of the Gram‐negative bacterial cell wall. Under normal circumstances, while LPS is rarely observed in the serum, it is present in high concentrations in the gut due to the presence of the intestinal flora. However, LPS can enter the circulation when damage to the intestinal barrier occurs, resulting in increased LPS levels in the serum. LPS can have adverse effects on the immune system and lead to excessive inflammatory responses, potentially causing organ dysfunction and injury. TLRs are pattern recognition receptors that are responsible for regulating cell survival and proliferation, as well as initiating inflammatory responses in various biological settings. Due to its critical role in identifying LPS, TLR4 has been widely investigated in studies on the development of human diseases. It is well‐established that TLR4 can trigger the activation and nuclear translocation of its downstream protein NF‐κB, a key transcription factor that promotes the expression of pro‐inflammatory cytokines and exacerbates inflammatory reactions. TLR4 has been found to be significantly upregulated in circulating mononuclear cells from patients with IgAN, and this increased expression is correlated with proteinuria and heavy microscopic hematuria [37]. TLR4 participates in IgA‐mediated mesangial cell activation and injury by inducing the release of pro‐inflammatory cytokines [38]. As recently reported, either silencing of TLR4 or inhibition of NF‐κB can reduce secretory IgA‐induced synthesis of TNF‐α, IL‐6, and MCP‐1 by human renal mesangial cells, indicating the crucial role of the TLR4/NF‐κB signaling pathway in mediating the kidney injury associated with IgAN [39]. It has also been found that forsythiaside A can alleviate inflammation and epithelial barrier damage in LPS‐induced macrophages and TNF‐α‐induced lung/colon epithelial cells, as well as in the lung and colon tissues of mice with LPS‐induced acute lung injury through suppression of the TLR4/MAPK/NF‐κB and NF‐κB/MLCK/MLC2 signaling pathways [20]. The present findings showed that forsythiaside A reduced LPS levels in the sera and TLR4, p‐NF‐κB p65, and p‐IκBα protein levels in the kidneys and intestines of IgAN rats.

However, this study has several limitations that warrant future investigation. First, the IgAN pathogenesis is complex, involving a variety of interactions among multiple renal cell types (such as mesangial cells, glomerular endothelial cells, podocytes, and tubular epithelial cells), which jointly drive damage to the glomeruli and tubulointerstitial tissues. The present findings provide only preliminary evidence that forsythiaside A can ameliorate pathological renal injury and inflammation in IgAN rat models, and did not undertake an in‐depth exploration of the underlying molecular and cellular mechanisms of IgAN. Second, although the study demonstrated the effectiveness of forsythiaside A in alleviating intestinal epithelium dysfunction and kidney injury in IgAN rats, the effects of forsythiaside A on the gut microbiota were not investigated; this warrants further investigation and would contribute to the understanding of the crosstalk among the gut, immune system, and kidney in IgAN. Third, the bioavailability of forsythiaside A is relatively low, with a content of 0.5% observed in rat plasma after oral administration; this may result from possible hydrolysis in the gastrointestinal tract, poor permeability through the intestinal epithelial membrane, and first‐pass effects in the liver [40]. A combined assessment of the physicochemical properties (such as lipophilicity, molecular weight, and solubility) and pharmacokinetic characteristics of forsythiaside A could be used to select appropriate methods to improve the oral bioavailability of forsythiaside A. For example, the inclusion of forsythiaside in nanoparticles, nanoemulsions, or liposomes could increase its solubility and intestinal permeability, and reduce the likelihood of gastrointestinal hydrolysis. Fourth, the present findings only revealed that the renoprotective effects of forsythiaside A in IgAN rats were linked with inhibition of the TLR4/NF‐κB pathway, and TLR4 agonists were not used to verify the role of the TLR4/NF‐κB signaling in the effects of forsythiaside A on IgAN progression. Furthermore, the potential involvement of other signaling pathways in the renoprotective effects of forsythiaside A in IgAN remains unknown and warrants further research.

**FIGURE 6:**
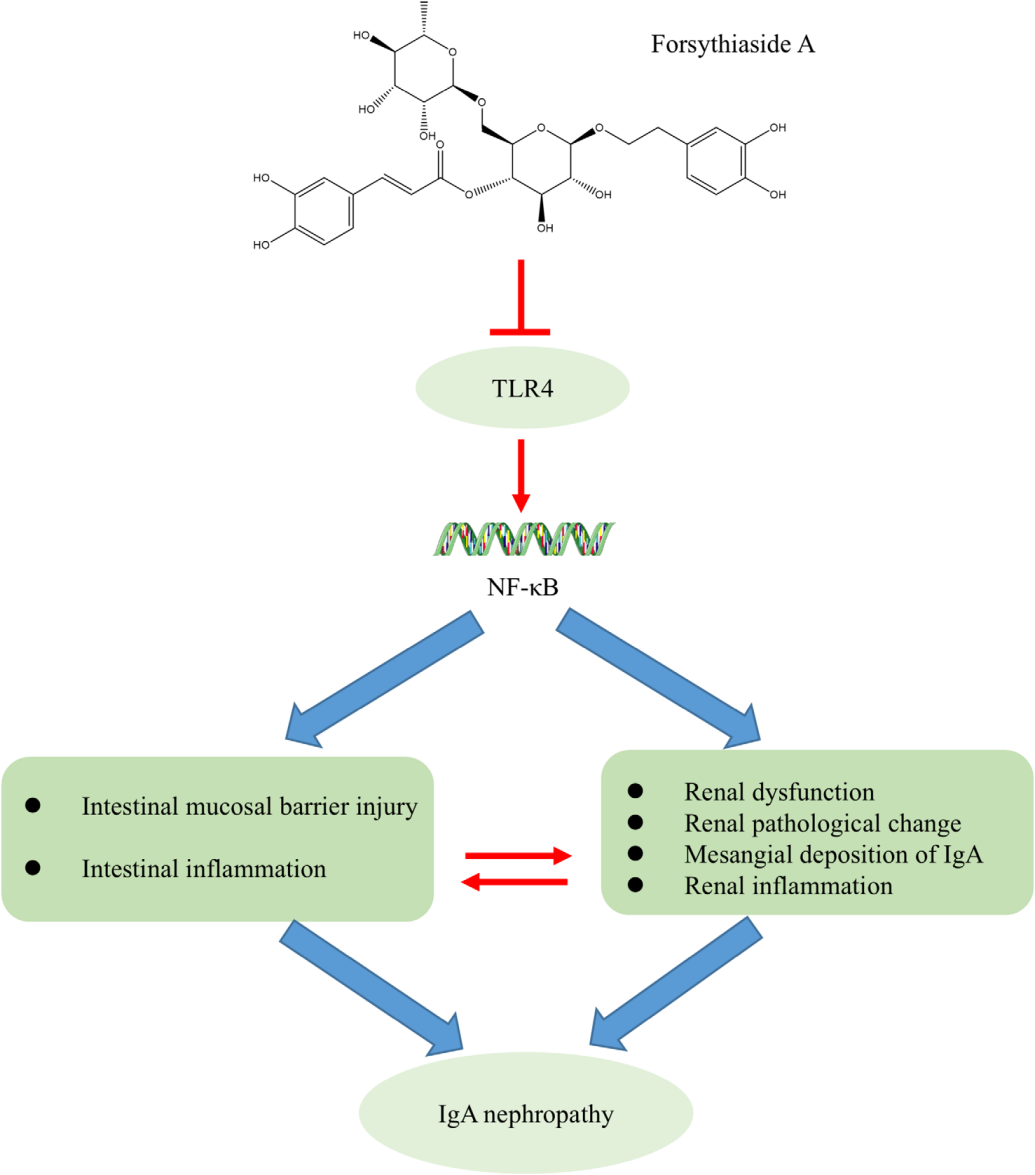
Graphical abstract. Forsythiaside A protects against both renal and intestinal injury during the progression of IgA by inhibiting the TLR4/NF‐κB pathway.

In conclusion, the study identified the therapeutic effects of forsythiaside A in protecting against IgAN in rat models by ameliorating inflammation and injury to the intestinal barrier. Notably, this is the first demonstration of the involvement of the TLR4/NF‐κB signaling pathway in the renoprotective effects of forsythiaside A in the treatment of IgAN (Figure 6). The findings suggest the potential of forsythiaside A in the development of novel treatments for IgAN.

## Ethics Statement

All animal experiments were ethically approved by the Institutional Animal Care and Use Committee of Wuhan Myhalic Biotechnology Co. Ltd. (approval number: HLK‐202403057; approval date: March 5, 2024).

## Conflicts of Interest

The authors declare no conflicts of interest.

## Data Availability

The data that support the findings of this study are available from the corresponding author upon reasonable request.
